# Rehabilitation Strategies Following Isolated Meniscal Repair: A Systematic Review of Protocols and Outcomes

**DOI:** 10.3390/jcm15041616

**Published:** 2026-02-19

**Authors:** Waleed Albishi, Ibraheem Al Yami, Abdullah Alyami, Omar A. Aldosari, Sarah AlJasser

**Affiliations:** 1 Department of Orthopedic Surgery, College of Medicine, King Saud University, Riyadh 11362, Saudi Arabia; 2 Department of Orthopedic Surgery, King Khalid Hospital, Ministry of Health, Najran 66262, Saudi Arabia; 3 Medical Rehabilitation Sciences Department, Applied Medical Sciences College, Najran University, Najran 61441, Saudi Arabia

**Keywords:** meniscal repair, rehabilitation protocols, knee injury, physical therapy, early weight-bearing, range of motion, functional outcomes, systematic review

## Abstract

**Background:** Meniscal injuries are common athletic injuries, and isolated meniscal repair is a critical procedure for restoring knee function. However, rehabilitation protocols after meniscal repair remain controversial. This systematic review aimed to evaluate rehabilitation protocols to determine the best strategies for enhancing recovery following isolated meniscal repair. **Objectives:** Analyze current rehabilitation protocols following isolated meniscal repair, focusing on the efficacy of approaches in improving functional outcomes and reducing recovery time. This study also aims to identify gaps in the existing literature and provide recommendations for future studies. **Data sources:** Search was conducted using PubMed, Scopus, and Web of Science databases, covering studies published between May 2015 and May 2024. Inclusion criteria: Studies reporting on isolated meniscal repair with defined postoperative rehabilitation protocols and quantifiable outcome measures. Data extraction: Focused on patient demographics, meniscal tear types, repair techniques, and rehabilitation outcomes. The methodological quality of the included studies was assessed, and narrative synthesis was conducted. **Results:** The review included 13 studies with significant variability in rehabilitation protocols and outcomes. Early weight-bearing and range of motion exercises have been associated with improved recovery in some studies for stable meniscal tears. However, conservative approaches have better outcomes in patients with complex tears. The use of adjunctive therapies such as blood flow restriction training has demonstrated potential in enhancing muscle preservation and overall recovery. **Conclusions:** Rehabilitation protocols after meniscal repair surgery should be individualized. Although innovative protocols show promise, further research is needed to standardize rehabilitation approaches and optimize long-term outcomes.

## 1. Introduction

The meniscus is a crucial component of the knee joint that is prone to injury, particularly in athletes and active individuals [1]. Without the menisci, knee joint function is severely compromised, leading to progressive degeneration of the articular cartilage [2]. While some meniscal tears may be amenable to surgical repair, the optimal rehabilitation protocols to maximize the chances of successful healing have not been well-established.

Meniscal tears are the most common knee injuries, and the treatment approach depends on the pattern and location of the tear [3]. Longitudinal tears in the vascular zone of the meniscus can often be repaired, with success rates as high as 87% [4]. However, tears in the avascular zone typically require partial meniscectomy, which increases the risk of subsequent osteoarthritis [4]. Epidemiological evidence from an unselected adult population shows that sport-related meniscal injuries account for 32.4% of cases (mean age 33 years), whereas non-sporting injuries represent 38.8% (mean age 41 years)—with 71.9% occurring during activities of daily living—and 28.8% of tears occur without an identifiable injury mechanism (mean age 43 years), highlighting substantial differences in age distribution, injury context, and functional demands across meniscal injury populations [5].

To address this challenge, researchers have been exploring innovative rehabilitation protocols that may improve outcomes for patients undergoing isolated meniscal repair. One such approach involves an accelerated rehabilitation program that emphasizes early weight-bearing and range-of-motion exercises, in contrast to the more traditional, more conservative protocols that often involve extended periods of immobilization [3,4].

Innovative rehabilitation protocols for isolated meniscal repair are evolving; however, there remains a lack of consensus on the optimal approach. The variability in rehabilitation strategies is influenced by the type and stability of meniscal tears. For instance, tears retaining hoop integrity often allow for accelerated rehabilitation, with partial weight bearing (PWB) permitted immediately and full weight bearing (FWB) at approximately four weeks postoperatively. In contrast, tears without hoop integrity necessitate a more conservative approach, delaying PWB to four weeks and FWB to six weeks [6,7].

The French Society of Arthroscopy (SFA) study highlighted that immediate weight bearing and the use of braces are associated with higher failure rates, suggesting that a cautious approach may be beneficial, especially for large or circumferential fiber tears [8]. Despite these findings, there is no uniform agreement on the best practices, and the effectiveness of rehabilitation protocols can vary significantly based on the degree and type of meniscal injury [9]. Furthermore, adjuncts such as synovial rasping and biological augmentation are common, although the latter are less frequently employed [1].

Although accelerated rehabilitation protocols are gaining popularity, concerns remain regarding their suitability for all types of meniscal repair, particularly unstable tears. This systematic review aimed to analyze the existing literature on rehabilitation protocols following isolated meniscal repair. We anticipate variability in existing protocols and a lack of consensus on standardized, universally applicable guidelines. By synthesizing the current evidence, this review highlights areas requiring further research to establish evidence-based protocols and improve patient outcomes.

## 2. Materials and Methods

### 2.1. Study Design and Reporting Standards

This systematic review was conducted in accordance with the Preferred Reporting Items for Systematic Reviews and Meta-Analyses (PRISMA) guidelines [10] [Table S1]. The review aimed to evaluate and synthesize postoperative rehabilitation protocols following isolated meniscal repair, with particular emphasis on weight-bearing progression, range-of-motion advancement, and functional outcomes. The population comprised adult patients undergoing isolated meniscal repair, the intervention encompassed postoperative rehabilitation strategies (including weight-bearing progression, range-of-motion advancement, bracing, and adjunctive therapies), and the outcomes included functional recovery, patient-reported outcome measures, and return-to-activity indicators. Given the heterogeneity of rehabilitation approaches and study designs, no single comparator was predefined, and comparisons were instead addressed through narrative synthesis.

### 2.2. Literature Search Strategy

A comprehensive literature search was conducted across the PubMed, Scopus, and Web of Science databases. Studies published between May 2015 and May 2024 were considered eligible. The search strategy combined keywords and MeSH terms related to meniscal repair and rehabilitation, including: “meniscus repair” OR “meniscal repair” AND “rehabilitation” OR “physical therapy” OR “exercise therapy” OR “recovery” AND “protocol” OR “guideline” OR “program.”

Reference lists of included articles and relevant review papers were manually screened to identify additional eligible studies not captured by the electronic search.

### 2.3. Eligibility Criteria

Studies were included if they:

1. Reported outcomes following isolated meniscal repair;
2. Described clearly defined postoperative rehabilitation protocols;
3. Provided quantifiable outcome measures, including functional scores, return-to-activity outcomes, or validated patient-reported outcome measures.

Studies were excluded if they involved:

1. Concomitant ligament reconstruction (e.g., ACL or PCL);
2. Meniscal root repair, meniscal transplantation, or cartilage repair procedures;
3. Case reports, conference abstracts, or review articles;
4. Insufficient description of rehabilitation protocols or outcome measures.

### 2.4. Study Selection Process

Two independent reviewers screened titles and abstracts of all retrieved records. Full-text articles were then assessed for eligibility based on the predefined inclusion and exclusion criteria. Disagreements were resolved through discussion or consultation with a third reviewer. The study selection process is illustrated using a PRISMA flow diagram.

### 2.5. Data Extraction

Data were extracted using a standardized form and included: study design, sample size, patient demographics, meniscal tear characteristics, repair technique, postoperative rehabilitation protocol details (including weight-bearing status, range-of-motion restrictions, bracing, and adjunctive therapies), and reported outcome measures. Data extraction was performed independently by two reviewers to ensure accuracy.

### 2.6. Methodological Quality Assessment

The methodological quality of randomized controlled trials was assessed using the Physiotherapy Evidence Database (PEDro) scale, while non-randomized and quasi-experimental studies were evaluated using the Joanna Briggs Institute (JBI) Critical Appraisal Checklist. Quality assessments were conducted independently by two reviewers, with discrepancies resolved by consensus.

### 2.7. Data Synthesis

Due to substantial heterogeneity in study design, rehabilitation protocols, outcome measures, and patient populations, a formal meta-analysis was not performed. Instead, a narrative synthesis was undertaken, with studies grouped according to rehabilitation strategy (accelerated versus restricted protocols), tear stability, and weight-bearing and range-of-motion progression. This approach allowed clinically meaningful comparison of rehabilitation principles while avoiding inappropriate quantitative pooling.

## 3. Results

**Figure 1:**
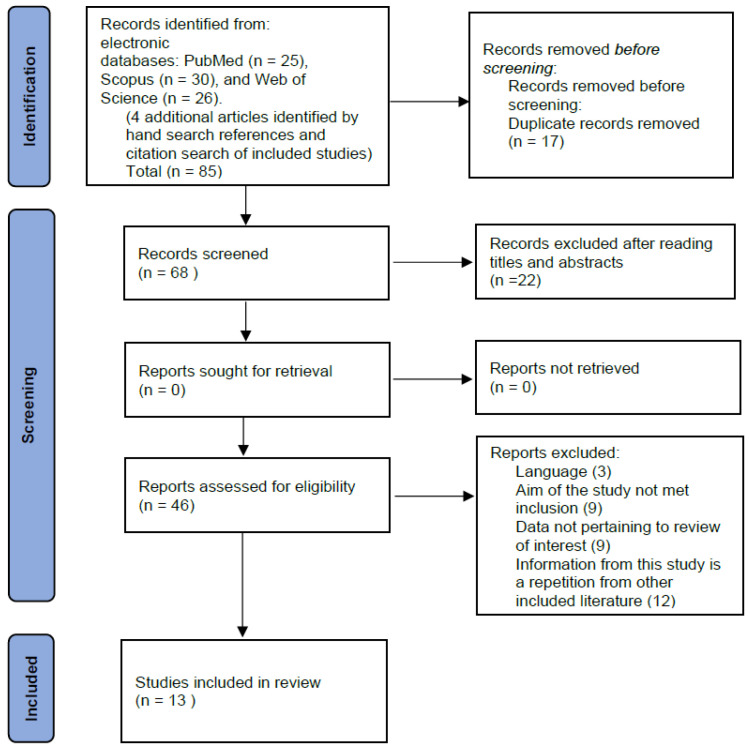
PRISMA flow diagram.

The initial literature search and protocol are presented in the flow diagram (Figure 1). The search process identified a total of 85 records, with 81 sourced from electronic databases—PubMed (n = 25), Scopus (n = 30), and Web of Science (n = 26)—and an additional 4 articles identified through hand searching references and citation searches of included studies. After removing 17 duplicate records, 68 records were screened based on their titles and abstracts. Of these, 22 records were excluded due to irrelevance.

The remaining 46 full-text articles were assessed for eligibility. Of these, 33 articles were excluded for the following reasons: language barrier (n = 3), not meeting the study’s inclusion criteria (n = 9), data not pertaining to the review’s focus (n = 9), and information from this study is a repetition of other included literature (n = 12). Ultimately, 13 studies were considered eligible and included in this review.

Table 1 provides a detailed overview of the study designs, sample sizes, patient demographics, types of meniscal tears, repair techniques, and primary outcome measures across the various studies focused on meniscal repair.

The study by Tahami et al. [1], a prospective cohort study involving 43 patients with medial meniscal posterior root tears (MPRT), utilized the loop-post construct technique for repair. The results showed that, after two years, a substantial proportion of patients achieved excellent (37.2%) or good (41.8%) functional outcomes, as measured by Lysholm knee scores. However, a smaller percentage of patients experienced fair (16.2%) or poor (4.6%) outcomes, highlighting the variability in patient recovery.

Skou et al. [12] conducted a multicentre, parallel-group RCT with 140 patients, although the specific tear types and exact repair technique were not detailed. The study reported significant improvements in the KOOS scores for both treatment groups, suggesting that the arthroscopic repair technique, regardless of its specifics, effectively enhanced patient outcomes.

Favreau et al.’s [8] retrospective study of 367 patients highlighted the differences in outcomes based on post-surgery weight-bearing practices. The study found that patients who deferred weight bearing had better functional outcomes, as indicated by a higher TEGNER score of 6.5 than those who began weight bearing immediately with a score of 5.4. Additionally, the KOOS Quality of Life score was notably higher in patients who did not use a brace postoperatively, suggesting that these factors might contribute to an improved quality of life.

Van De Graaf et al. [13] provided insights from a multicentre RCT involving 289 patients with non-obstructive meniscal tears. This study compared outcomes between patients who underwent arthroscopic partial meniscectomy (APM) and those who received physical therapy (PT). Both groups showed significant improvements in knee function, with APM leading to a greater mean improvement of 26.2 points in the IKDC score compared to 20.4 points in the PT group, indicating that surgical intervention may offer more substantial functional gains.

Another RCT by Skou et al. [14], involving 107 patients, assessed the outcomes of meniscectomy and meniscorraphy. Both groups demonstrated clinically relevant improvements in KOOS 4 scores at the 12-month follow-up, along with gains in physical performance measures, such as muscle strength and functional capacity, further supporting the effectiveness of surgical and non-surgical rehabilitation strategies.

Jahan et al. [15] conducted an experimental study of 38 patients, focusing on traumatic and degenerative meniscal tears treated with partial meniscectomy. The results indicated significant improvements in range of motion (ROM), muscle strength, and overall functional capacity, emphasizing the benefits of targeted rehabilitation.

Lastly, a feasibility study by Skou and Thorlund [12,14,16] involving six patients with MRI-confirmed meniscal injuries reported significant improvements in KOOS scores following partial meniscectomy, reinforcing the positive outcomes associated with this approach.

Overall, the studies summarized in Table 1 demonstrate a wide range of meniscal repair techniques and rehabilitation protocols, each contributing to varying degrees of functional improvement and patient satisfaction. These findings underscore the importance of individualized treatment plans and the potential benefits of weight-bearing and bracing in certain cases to optimize recovery and quality of life.

Table 2 summarizes various meniscal repair and rehabilitation protocols, highlighting the specific advice given to patients regarding range of motion and weight-bearing, along with the outcomes measured.

Koch et al. [17] studied 54 protocols for meniscal repair and found that early postoperative restrictions on range of motion were common. Full weight-bearing typically began around the second postoperative week, with specific advice varying based on the type of meniscal therapy. The study noted that the mean time to initiate full weight bearing was approximately 3.9 weeks, and following these guidelines led to positive outcomes, particularly in terms of ROM and weight-bearing at six weeks post-surgery.

Antao et al. [18] focused on 52 patients with meniscal root tears treated arthroscopically. Their protocol advised patients to remain non-weight-bearing for the first six weeks, with restricted knee flexion (0–90 degrees) for the first two weeks, gradually increasing as tolerated. Weight bearing began after six weeks with the aid of crutches, which were later weaned off. The study reported significant improvements in both Visual Analog Scale (VAS) and Lysholm Knee Scores over six months, indicating that this conservative approach effectively managed pain and improved knee function.

You et al. [19] analyzed 11 studies with 612 patients divided into accelerated and restricted rehabilitation groups. The accelerated group, which began immediate mobilization and weight bearing, showed a significant improvement in the Lysholm score compared to the restricted group, which followed a slower progression. These findings suggest that an accelerated rehabilitation protocol may lead to better self-reported function post-surgery, although the authors recommend further large randomized trials to confirm the optimal rehabilitation strategy.

Tahami et al. [11] examined 43 patients with medial meniscal root tears, where ROM exercises were initiated two weeks post-operation, aiming for 90 degrees of flexion by the sixth week. Partial weight-bearing exercises were started within the first two weeks, with full weight-bearing permitted after six weeks. Significant improvements in Lysholm knee scores were observed at the two-year follow-up, although no significant improvement was noted in the “Using cane or crutches” outcome, suggesting that while the overall recovery was positive, some functional limitations remained.

Overall, Table 2 illustrates the diverse range of rehabilitation protocols following meniscal repair and emphasizes the importance of individualized advice on weight bearing and range of motion to optimize recovery outcomes. Each study supports the idea that tailored rehabilitation strategies are crucial for achieving the best possible functional improvement after meniscal repair.

Table 3 focuses on the outcomes of dual restriction protocols, which involve specific restrictions on weight-bearing and range of motion following meniscal repair. The table highlights the impact of these restrictions on patient outcomes, particularly in terms of functional recovery and muscle preservation.

Jakobsen et al. [20] studied 40 patients with weight-bearing restrictions after knee cartilage or meniscus repair. This study introduced blood flow restriction–low load strength training (BFR-LLST) as a method to prevent disuse thigh muscle atrophy during these restrictions. While the exact duration of ROM restriction was not specified, the study recorded significant improvements in thigh circumference, indicating that BFR-LLST effectively mitigated muscle loss during the recovery period. Additionally, significant improvements were observed across the various KOOS subscales, reflecting better symptoms, pain management, and daily function. The Patient-Specific Functional Scale (PSFS) also showed an enhanced ability for patients to perform important activities, with scores improving significantly from baseline.

Koch et al. [17], in a study involving 54 patients, reported that weight-bearing was restricted for approximately 3.9 weeks, while range of motion was restricted for six weeks following meniscal repair. The study suggested that early functional rehabilitation led to improved outcomes, particularly after partial meniscectomy, with significant differences in recovery measures compared to traditional meniscal repair techniques.

Overall, Table 3 emphasizes the positive impact of dual restriction protocols on patient outcomes after meniscal repair. The studies highlighted in the table suggest that carefully managed restrictions on weight-bearing and range of motion, combined with innovative rehabilitation techniques, such as BFR-LLST, can lead to significant improvements in muscle preservation, functional recovery, and overall patient satisfaction. These findings reinforce the importance of tailoring postoperative protocols to enhance recovery and optimize long-term outcomes in patients undergoing meniscal repair.

## 4. Discussion

The novelty of the present review lies not only in summarizing existing rehabilitation protocols but in synthesizing protocol characteristics in relation to tear stability, repair integrity, and postoperative loading tolerance, thereby translating heterogeneous evidence into clinically meaningful guidance. By contrasting accelerated and restricted rehabilitation strategies across different meniscal repair contexts, this review provides a framework to assist clinicians in individualizing postoperative decision-making, particularly with respect to timing of weight-bearing, progression of range of motion, and incorporation of adjunctive interventions such as blood-flow-restriction training. Rather than proposing a single universal protocol, this synthesis highlights decision nodes that can guide rehabilitation planning when balancing repair protection with functional recovery in routine clinical practice.

One of the most significant insights from this review is the growing support for early weight-bearing and range of motion (ROM) exercises to enhance recovery outcomes. When considered specifically as a full weight-bearing (FWB) strategy, this approach has primarily been applied in the context of stable meniscal repairs with preserved hoop stress. Koch et al. provided compelling evidence that early postoperative restrictions on ROM, coupled with a gradual initiation of full weight-bearing around 3.9 weeks post-surgery, led to positive outcomes in patients who underwent meniscal repair [17,18]. These findings suggest that, for certain types of meniscal repair, particularly those with intact hoop stress, an accelerated rehabilitation protocol may be advantageous. The ability to engage in full weight-bearing earlier in the recovery process could potentially reduce the recovery time and improve overall knee function [21].

However, the potential risks associated with accelerated rehabilitation protocols should be carefully considered. Immediate or poorly selected FWB strategies may expose the repair site to excessive mechanical loading, particularly in complex tear patterns. The retrospective study by Favreau et al. [8] highlighted the dangers of immediate weight-bearing and brace use in patients with more complex or unstable meniscal tears. The study reported higher failure rates in these patients, suggesting that, in cases of large or circumferential fiber tears, a more conservative approach might be necessary to protect the integrity of the repair. These findings suggest that, in cases of large or circumferential fiber tears, a more conservative approach emphasizing partial weight-bearing (PWB) may be necessary to protect the integrity of the repair. This observation aligns with the broader clinical consensus that tear morphology, location, and stability significantly influence the appropriate postoperative loading strategy [22].

Skou et al. [16] further contributed to this discussion by examining both surgical and nonsurgical rehabilitation strategies. Their multicenter RCT demonstrated significant improvements in KOOS scores, muscle strength, and functional capacity in both the surgical and exercise-based rehabilitation groups. Importantly, these findings support the concept that weight-bearing progression—whether FWB or PWB—should be integrated within a broader, patient-specific rehabilitation strategy, rather than dictated solely by surgical intervention. While surgery may offer more substantial immediate improvements in knee function, well-designed rehabilitation programs can yield meaningful long-term benefits across different loading strategies. This reinforces the need to tailor rehabilitation protocols not only to surgical technique but also to patient-specific recovery potential and activity level.

The use of adjunctive rehabilitation techniques has also emerged as a key theme of this review. Jakobsen et al.’s exploration of Blood Flow Restriction—Low Load Strength Training (BFR-LLST) provided valuable insights into how such methods can enhance recovery during periods of restricted weight-bearing and ROM [20,21]. Adjunctive strategies such as BFR-LLST are particularly relevant in PWB protocols, as they help prevent disuse muscle atrophy. The BFR-LLST helps maintain muscle mass and strength, which are crucial for supporting knee function and stability during the early stages of recovery. This approach could be particularly beneficial for patients who are unable to engage in full weight-bearing activities because of the nature of their meniscal tears or repair technique [23].

Interestingly, this review also highlighted the role of psychological and patient-specific factors in the success of rehabilitation protocols. For instance, Antao and Desouza’s study on patients with meniscal root tears demonstrated that adherence to a conservative rehabilitation protocol, involving non-weight-bearing for six weeks followed by gradual flexion and weight-bearing, resulted in significant pain reduction and functional improvement [18,19]. The success of this approach likely reflects not only the physical benefits of the protocol, but also the importance of patient education, motivation, and adherence to prescribed rehabilitation regimens. These factors are critical for ensuring that patients do not prematurely stress the repaired meniscus, which could compromise healing.

Moreover, the variability in outcomes across different studies reflects the ongoing debate in the field regarding the optimal balance between the protection of the repair site and the need for early mobilization to prevent joint stiffness and muscle atrophy. For instance, You et al. found that patients in the accelerated rehabilitation group, who began immediate mobilization and weight bearing, showed significantly better self-reported functional outcomes than those in the restricted group, which followed a more gradual progression [16,20]. However, this study also highlights the need for larger randomized trials to confirm these findings and to establish clear guidelines on which patient populations would benefit most from accelerated versus conservative approaches.

The findings of these studies collectively suggest that while accelerated rehabilitation protocols offer promise, particularly for stable meniscal tears, their application must be carefully considered on a case-by-case basis. Patients with more complex or unstable tears may require more conservative rehabilitation strategies to prevent repair failure and to ensure long-term joint health. Furthermore, the integration of adjunctive therapies such as BFR. LLST may play a crucial role in enhancing recovery, especially in maintaining muscle mass and strength during periods when traditional weight-bearing activities are not possible.

Despite the nature of this study, several limitations should be acknowledged. First, the heterogeneity of the included studies, particularly in terms of patient demographics, types of meniscal tears, and specifics of surgical and rehabilitation protocols, makes it challenging to draw definitive conclusions. The variability in outcome measures and lack of standardized protocols across studies further complicate the comparison of results. Additionally, the reliance on retrospective studies and the inclusion of studies with small sample sizes may introduce bias, limiting the generalizability of the findings. Another limitation is potential publication bias, as studies with positive outcomes are more likely to be published, potentially skewing the conclusions of the review.

Future research should focus on conducting large-scale randomized controlled trials (RCTs) to establish more definitive guidelines for postoperative rehabilitation following meniscal repair. These studies should aim to standardize rehabilitation protocols across different types of meniscal tears and patient populations, allowing for more accurate outcome comparisons. Additionally, research should explore the long-term effects of accelerated versus conservative rehabilitation strategies, particularly in terms of preventing re-injury and the development of osteoarthritis. The role of adjunctive therapies, such as blood flow restriction training and biological augmentation, should be further investigated to determine their efficacy in enhancing recovery and long-term knee function. Finally, studies that incorporate patient-reported outcomes and quality of life measures are crucial in assessing the overall success of rehabilitation protocols from the patient’s perspective.

## 5. Conclusions

In conclusion, the variability in rehabilitation protocols and outcomes observed in this review underscores the lack of consensus regarding a standardized approach to postoperative care following isolated meniscal repair. Rather than supporting uniform, time-based protocols, the synthesized evidence favors a decision-based rehabilitation approach in which postoperative strategies are guided by tear morphology, repair stability, and biological healing considerations. While innovative rehabilitation strategies such as early weight-bearing and the use of adjunctive therapies show promise—particularly in stable repairs—more conservative progression remains appropriate for complex or biologically vulnerable tears to protect repair integrity. Adjunctive interventions, including blood-flow-restriction training, may further support functional recovery during periods of restricted loading. As the field continues to evolve, high-quality studies are needed to refine these strategies and define the patient populations most likely to benefit, ultimately enabling the development of evidence-based guidelines that integrate tear characteristics, patient factors, and surgical techniques to optimize clinical outcomes following meniscal repair.

## Figures and Tables

**Table 1:** Study Details and Patient Demographics.

Variable/Study	Tahami et al. [10]	Skou et al. [11]	Favreau et al. [7]	Van De Graaf et al. [12]	Skou et al. [13]	Jahan et al. [14]	Skou et al. [15]
Design of study	Prospective cohort study	Multicentre, parallel-group RCT	Retrospective	Multicentre RCT	RCT	Experimental	Feasibility study
Sample size	43	140	367	289	107	38	6
Age, y, Mean (Range)	53 (18–56)	29 (18–40)	Not reported	58 (45–70)	29 (18–40)	28 (18–34)	23 (18–40)
Meniscal tear type	Medial meniscal posterior root tear (MPRT)	Types of tears not mentioned	Stable and unstable tear	Non-obstructive meniscal tears	Specific types not mentioned	Traumatic and degenerative	Meniscal injury confirmed by MRI
Repair technique/intervention	Loop-post construct technique	Arthroscopic repair, exact technique not mentioned	Not specify the exact technique	Partial meniscectomy	Meniscectomy, Meniscorraphy	Partial meniscectomy	Partial meniscectomy
Primary outcome measure	Two-year functional outcomes were categorized as excellent in 16 (37.2%), good in 18 (41.8%), fair in 7 (16.2%), and poor in 2 (4.6%) patients based on the total Lysholm knee scores	KOOS score significantly improve in both groups.	TEGNER score was significantly higher in the non-weight bearing group (6.5) compared to the immediate weight bearing group (5.4), suggesting better functional outcomes for those who deferred weight bearing. KOOS Quality of Life (QOL) score was also higher in the group without a brace (82.2) compared to those who wore a brace (66.8), indicating that not using a brace may lead to improved quality of life outcomes post-surgery	IKDC In the arthroscopic partial meniscectomy (APM) group, knee function improved from a baseline score of 44.8 points to 71.5 points, resulting in a mean difference of 26.2 points (95% CI, 23.2 to 29.3) In the physical therapy (PT) group, knee function improved from 46.5 points at baseline to 67.7 points, with a mean difference of 20.4 points (95% CI, 17.5 to 23.2)	Both groups (surgery and exercise) experienced clinically relevant improvements in KOOS 4 scores at the 12-month follow-up, indicating positive outcomes for both treatment strategies. Improvements were also noted in physical performance measures, including isometric leg press muscle strength and functional tests like knee bends and hops, assessed at various intervals (3 and 12 months)	ROM, muscle strength and functional capacity significantly improved.	Significant improvement in KOOS

**Table 2:** Meniscal Repair and Rehabilitation Protocols and Their Outcomes.

Variable/Study	Koch et al. [16]	Antao et al. [17]	You et al. [18]	Tahami et al. [10]
Study population	54 protocols for meniscus repair	52 patients with meniscal root tear treated by arthroscopic repair	11 studies with 612 patients analyzed. - 4 studies in accelerated group, 7 in restricted group.	- 43 consecutive patients with medial meniscal root tears studied - 30 patients underwent surgical repair of meniscal root tears
Range of motion advice	Early postoperative restrictions on range of motion after meniscus repair.	0–90° flexion for first 2 weeks, after 2 weeks flexion increased as tolerated	Accelerated program: Immediate mobilization; Restricted program: 2-week immobilization	Range of motion exercises started 2 weeks post-op. Goal: Reach 90 degrees flexion by 6th week post-op.
Weight bearing advice	Full weight bearing starts from the second postoperative week. Weight bearing advice varies based on meniscus therapy type.	Non-weight bearing for 6 weeks, then weight bearing using crutches	Accelerated: Immediate weight-bearing after meniscus suture. Restricted: Partial weight-bearing for at least 3 weeks post-surgery.	- Partial weight-bearing exercises started during the first two weeks. - Full weight-bearing permitted after six weeks post-surgery.
Specific advice for patients	Follow postoperative weight bearing and range of motion recommendations. Engage in physiotherapy and rehabilitation training as advised.	- Follow post-operative physiotherapy for optimal knee function recovery. - Meniscal root tears must be identified early for successful repair.	- Follow accelerated rehab for better self-reported function post-surgery. - Optimal program may be clarified by large randomized trials.	- Perform simple home-based exercises for rehabilitation. - Consider specialized physical therapy for better outcomes.
Outcome measure	ROM and weight bearing restricted for 6 weeks Result of this study indicated that the mean time for initiating full weight bearing after meniscus repair was around 3.9 weeks	The VAS pre-operative was 7.46, the VAS post-operative at 6 weeks was 4.23, the VAS post-operative at 3 months was 3.12 the VAS post-operative at 6 months was 1.19. The pre-op was 68.52, the Lysholm Knee Score post-op—6 weeks was 81.72, the Lysholm Knee Score post-op—3 months was 85.72, the Lysholm Knee Score post-op—6 months was 92.23	Accelerated rehabilitation group showed a significant improvement in the Lysholm score, with a mean difference of −4.66 (*p* = 0.02) compared to the restricted group, indicating better self-reported function	Significant improvements in the total Lysholm knee score were observed in both groups at the two-year follow-up, with a *p*-value of less than 0.05 indicating statistical significance. However, the item “Using cane or crutches” did not show significant improvement (*p* = 0.065)

**Table 3:** Outcome of Dual Restriction Protocol.

Variable/Study	Jakobsen et al. [19]	Koch et al. [16]
Sample size	40 patients	54
Weight bearing restriction	Patients had weight-bearing restrictions after knee cartilage or meniscus repair. BFR-LLST (Blood Flow Restriction—Low Load Strength Training) helped prevent disuse thigh muscle atrophy during restrictions.	Weight bearing restriction for 3.9 weeks
Range of motion restriction	The study does not specify an exact number of days for which the range of motion was restricted.	Range of motion restricted for 6 weeks
Improved outcome measure	Thigh Circumference: Significant increases were observed in the thigh circumference of the operated leg, with an average increase of 0.12 cm per week in the meniscus repair group. Significant improvements were recorded across various KOOS subscales, reflecting better symptoms, pain, and function in daily activities. Patient-Specific Functional Scale (PSFS): Patients reported enhanced ability to perform self-selected important activities, with scores improving significantly from baseline.	The study suggests that early functional rehabilitation leads to improved outcomes, particularly after partial meniscectomy, with significant differences in recovery measures compared to meniscus repair techniques.

## Data Availability

Not applicable.

## Supplementary Materials

The following supporting information can be downloaded at: https://www.mdpi.com/article/10.3390/jcm15041616/s1, Table S1: Prisma checklist.
